# Toward Better Control of Inclusion Cleanliness in a Gas Stirred Ladle Using Multiscale Numerical Modeling

**DOI:** 10.3390/ma11071179

**Published:** 2018-07-10

**Authors:** Jean-Pierre Bellot, Jean-Sebastien Kroll-Rabotin, Matthieu Gisselbrecht, Manoj Joishi, Akash Saxena, Sean Sanders, Alain Jardy

**Affiliations:** 1 Institut Jean Lamour—UMR 7198 CNRS/Université de Lorraine, CS 50840, 54011 Nancy CEDEX, France; jean-sebastien.kroll-rabotin@univ-lorraine.fr (J.-S.K.R.); matthieu.gisselbrecht@univ-lorraine.fr (M.G.); manoj.joishi@univ-lorraine.fr (M.J.); Alain.Jardy@univ-lorraine.fr (A.J.); 2 Laboratory of Excellence on Design of Alloy Metals for low-mAss Structures (Labex DAMAS), Université de Lorraine, 57073 Metz, France; 3 Department of Chemical & Materials Engineering, University of Alberta, Edmonton, AB T6G 1H9, Canada; asaxena@ualberta.ca (A.S.); sean.sanders@ualberta.ca (S.S.)

**Keywords:** steel ladle, non-metallic inclusions, simulation, aggregation

## Abstract

The industrial objective of lowering the mass of mechanical structures requires continuous improvement in controlling the mechanical properties of metallic materials. Steel cleanliness and especially control of inclusion size distribution have, therefore, become major challenges. Inclusions have a detrimental effect on fatigue that strongly depends both on inclusion content and on the size of the largest inclusions. Ladle treatment of liquid steel has long been recognized as the processing stage responsible for the inclusion of cleanliness. A multiscale modeling has been proposed to investigate the inclusion behavior. The evolution of the inclusion size distribution is simulated at the process scale due to coupling a computational fluid dynamics calculation with a population balance method integrating all mechanisms, i.e., flotation, aggregation, settling, and capture at the top layer. Particular attention has been paid to the aggregation mechanism and the simulations at an inclusion scale with fully resolved inclusions that represent hydrodynamic conditions of the ladle, which have been specifically developed. Simulations of an industrial-type ladle highlight that inclusion cleanliness is mainly ruled by aggregation. Quantitative knowledge of aggregation kinetics has been extracted and captured from mesoscale simulations. Aggregation efficiency has been observed to drop drastically when increasing the particle size ratio.

## 1. Introduction

A huge number of inclusions is generated in the secondary steelmaking processes mainly during the deoxidation steps. Non-metallic inclusions such as sulfide and oxide particles may have a detrimental impact on both formability and fatigue life. Furthermore, inclusion size distribution is particularly critical because large aggregates are the most harmful to mechanical properties [1]. Nowadays, the reduction of the weight of high performance steel parts without affecting their mechanical properties is a new challenge, which requires continuous improvement of metal cleanliness. 

**Figure 1:**
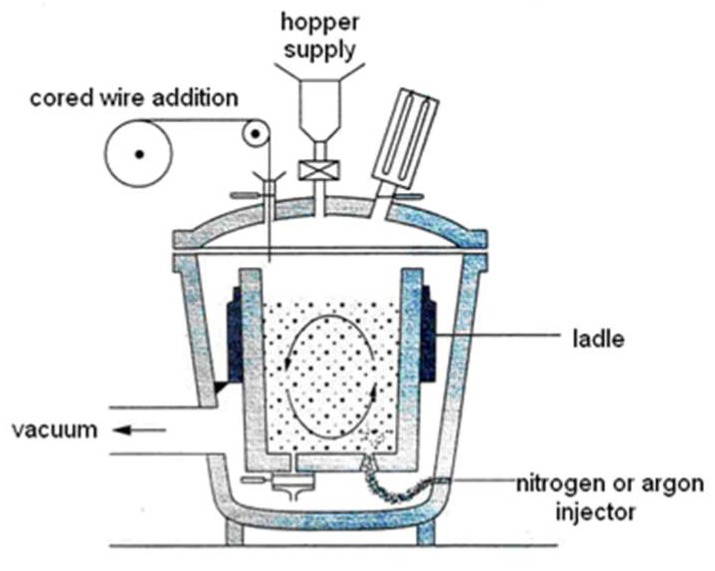
Schematic of a gas-stirred ladle.

Gas-stirred ladle treatment of liquid metal has long been recognized as the processing stage responsible for the inclusion population of specialty steels. As seen in Figure 1, argon gas is injected through one or more porous plugs at the bottom of the ladle, which leads to mixing of the liquid metal to achieve chemical homogeneity and entrapment of inclusions by the bubble swarm. The latter is well known as the flotation mechanism. Furthermore, the turbulence generated by the bubble plume enhances the probability of inclusion collision and enhances aggregation, which is an important mechanism for the evolution of the inclusion size distribution.

The ladle process is a very complex three-phase reactor, which has been the purpose of many modeling studies for the last two decades. Few cold or hot physical models [2,3] and numerous computational fluid dynamics (CFD) simulations have been undertaken to investigate the turbulent flow and mixing phenomena. The modeling of liquid-gas flows is treated in the literature using either a Euler-Euler method [4,5] or a Euler-Lagrange method [6,7]. Each approach has equally important advantages and drawbacks. Among the most sophisticated and recent approaches, the multiphase volume of the fluid (VOF) technique allowed Li et al. [8] to predict the opening of the slag due to bubbles called “open eye” (potentially leading to steel re-oxidation) and slag droplets entrainment (possibly generating large size defects). The behavior of the non-metallic inclusions in a gas-stirred ladle is usually modeled through a population balance method (PBM). The coupling of CFD and PBM techniques is now frequently adopted, which results in promising outcomes in terms of calculating the inclusion removal efficiency and evolution of particle size distribution (PSD) over processing time [9,10,11]. Recently, Lei et al. [12] proposed the application of a particle size grouping technique for solving the population balance equation across a very broad range of particle sizes (from nuclei of 0.1 nm up to large aggregates of the order of 100 μm in size) but with a noticeable discrepancy regarding the exact solution of Smoluchowski’s equation.

Unfortunately, all these sophisticated macroscopic models are based on modeled kinetics of turbulent aggregation and of aggregate restructuring (often described by a single morphological descriptor such as the fractal dimension) that are not capable of covering the exhaustive physics of inclusion interactions and are, in most cases, inadequate for oxide particles in a non-wetting liquid metal.

In the framework of project DAMAS (Design of Alloy Metals for low-mAss Structures), which is dedicated to the light-weighting of metallic materials, particular attention has been paid to the modeling of the behavior of inclusions. Specifically, an original multi-scale approach has been carried out. At the process scale, the evolution of the PSD is simulated by coupling a CFD calculation with a PBM integrating all mechanisms, i.e., flotation, aggregation, sedimentation, and capture at the top layer. This model takes into account the actual geometry of the full-scale industrial ladle and important operating conditions such as the gas flow rate. This 3D numerical tool is used to analyze the effect of successive different gas flow rates on the resulting population of non-metallic inclusions. At the inclusion scale, the simulations of fully resolved inclusions in hydrodynamic conditions represent those found in the ladle, which have been specifically developed. Such simulations are required in order to capture the local aggregation kinetics that impact the macroscopic behavior of inclusion. 

## 2. Methods and Computational Models

### 2.1. Mesoscale Modeling of Inclusion Behavior

Local dynamics at the inclusion scale are investigated through numerical simulations in which all scales of interest are resolved. The direct numerical simulation (DNS) of the flow uses a lattice Boltzmann method (LBM) [13,14] that is coupled with the dynamics of the solids using an immersed boundary method (IBM) [15], which is detailed by Saxena et al. [16]. Solids are discretized with marker points on their surfaces at which flow properties are interpolated from the surrounding LBM nodes. The momentum exchange between the solid and liquid phases is calculated for each marker point and is distributed back to the LBM nodes as a source term so that the flow undergoes stress from the action of solids. Similarly, the total force and torque acting on each solid object are obtained by adding all the contributions from all the marker points. The motion of the solids is then obtained by solving Newton’s equation and integrating it over time.

Such simulations of the flow and solid dynamics are fully deterministic. However, the information required in larger scale simulations is of statistical nature. This is why multiple simulations are required for all studies on inclusions and aggregate behavior.

#### 2.1.1. Aggregation Kinetics

Due to the strong non-wettability of non-metallic inclusions, colliding solids are assumed to form aggregates bonded by gas bridges. Therefore, in the present study, aggregation kinetics are not differentiated from collision kinetics. It is well established [17] that collision kinetics are driven by shear from which a collision frequency can be derived. This entails the number of events in a given time that could potentially lead to a collision between two solids. Hydrodynamic interactions between the solids will affect the outcome of such collision events and are usually represented by collision efficiency [18,19]. In the case of turbulence induced collision, kernels obtained by Saffman and Turner [20] yield very similar results to that derived by Smoluchowski with a shear value derived from the energy dissipation. It is, therefore, a common assumption that, as long as particles remain small when compared to the Kolmogorov turbulence length scale, their collision efficiency can be estimated from their interactions in a plane shear flow.

**Figure 2:**
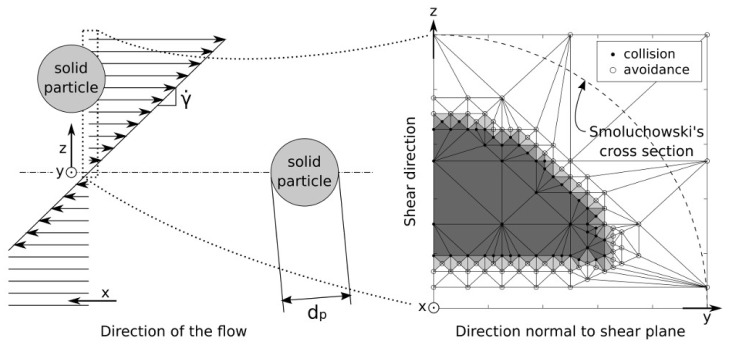
Simulation setup and example cross section derivation from 363 simulations of two identical spherical particles at Re*_γ_* = 0.213.

In this study, only spherical particles have been considered since non-metallic inclusions such as calcium aluminate found in the ladle simulated later are not completely solid but are rather highly viscous and have globular shapes. As illustrated in Figure 2, collision cross sections have been determined from simulations of particle pairs starting from various relative positions along the *y z* axes. Particles are initially far enough away from each other (along the *x* axis) and do not see the flow disturbance induced by each other. Then, the shear flow drives them closer and, depending on their initial relative positions, particles collide or avoid each other. The collision cross section is the set of all the initial positions in the upstream plane of one particle relatively to the other that lead to collision. It has been determined with an iterative process by identifying sets of relative positions leading to collision and avoidance. This procedure is illustrated in Figure 2 in which collision outcomes are represented for various upstream relative positions of two particles in which empty circles mark avoidance outcomes while black dots mark actual collisions. The upstream plane is iteratively subdivided in order to estimate the cross-section, which is represented by the gray surface.

The same procedure has been followed for several Reynolds numbers where the Reynolds number is defined by the equation below:

(1) Reγ˙=γ˙dp2υ

**Figure 3:**
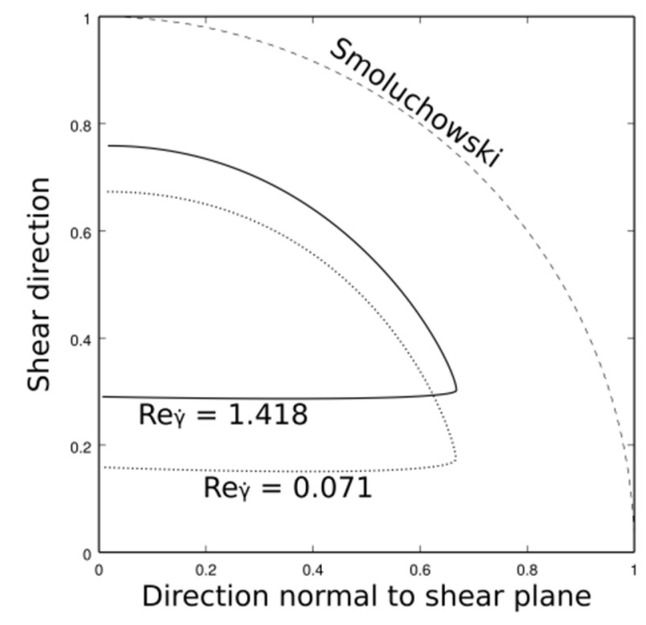
Evolution of the cross section with the shear-based Reynolds number for two spheres of the same size.

Cross-sections (surface $S_{Col}$) have been calculated (see Figure 3) for different Reynolds numbers and diameter ratios between interacting particles. Collision efficiencies are derived from these cross-sections by comparing the particle rate going through them with the Smoluchowski’s collision kernel.

(2) $$\eta =\frac{\iint_{S_{Col}}\dot{\gamma }zdydz}{\iint_{S_{Smo}}\dot{\gamma }zdydz}$$

#### 2.1.2. Aggregate Restructuring

A discrete element method (DEM) to account for non-hydrodynamic interactions between solids such as contact forces has also been used beside the LBM and IBM when studying deformable aggregate behaviors. Although this adds a new level of modeling in particle interactions, simulations remain deterministic and have to be repeated in order to extract statistical data. Therefore, a set of 10 aggregates with the same fractal dimension has been algorithmically created, exposed to various shear flows, and to various cohesive force intensities. The aggregate creation procedure [16] starts with a single particle and iteratively adds another one by picking the one location, which yields the fractal dimension closest to the target among a set of random possible locations in contact with other particles in the aggregate. Cohesive forces have been modeled by a normal force superposing a Van der Waals and born potentials [16] and by a tangential force in the form of a capped resistance to bending [21]. Aggregate morphological evolutions for different ratios of cohesive-to-shear forces have been investigated via numerical simulations.

### 2.2. Macroscale Modeling of the Steel Ladle

The approach adopted for reactor modeling is divided into two parts. The bubble plumes play an important role in metal bath mixing while, owing to their small weight fraction (<0.01%), the inclusions do not affect the flow. First, the two-phase turbulent flow of gas bubbles and liquid metal is simulated for the full 3D geometry of the ladle and a strong coupling is achieved between the liquid metal and the bubbles [7]. 

In a second step, the population balance equation is solved in each control volume by applying the cell-average technique proposed by Kumar [22], which is a variant of the fixed pivot method of Kumar and Ramkrishna [23]. This leads to a significant reduction in numerical diffusion. The solution to the system of resulting equations is based on the Fluent CFD V17.1 software in which a large number of user defined functions (UDF) has been incorporated.

#### 2.2.1. Hydrodynamic Simulation

The modeling of liquid-gas flows applied to ladle treatment of molten steel is treated using a Euler-Euler method. Following this approach, the dispersed phase (gas) is governed by a set of transport equations (continuity and momentum) similar to the equations applied for the continuous phase (liquid). Because of the strong turbulent nature of the fluid flow, a multiphase k-ε model has been selected in order to assess the effective viscosity in both phases. In this model, called the “two-fluid model,” the two sets of transport equations are coupled through the interaction force (*F_g_* = −*F_l_*) between the molten steel and the bubble swarm where *F_g_* is expressed as the sum of four contributions *F_gD_*, *F_gL_*, *F_gVM_*, and *F_kTD_*, which are, respectively, drag, lift, virtual mass, and turbulent dispersion. A detailed description and expression of these contributions are reported in Reference [7].

#### 2.2.2. Population Balance Modeling (PBM)

The population balance equation (PBE) is a generalized transport equation of the number density of a particle population. It accounts for various ways in which a population with a specific set of properties (here only the size of inclusions is considered) may appear or disappear [23]. The PBM treats both convective processes as well as the interaction at the individual particle scale within the particle population setting. The PBE may be solved using different numerical methods such as the Method of Moments (MOM) and the Class Method (CM). We have chosen the Class Method based on the discretization of the particle size distribution into M classes where *N_i_* is the number of inclusions of class “*i*” per unit volume of liquid metal or particles with their diameter comprised between *d_pi_* and *d_pi_*_+1_. The discretized PBE is given in Equation (3) where the left-hand side represents the macroscopic transport of inclusions and the right-hand side models mesoscopic phenomena such as bubble-inclusion (flotation *Z_bi_*) and inclusion-inclusion (aggregation *B_i_* − *D_i_*) interactions.

(3) $$\frac{\partial \alpha_{l}N_{i}}{\partial t}+\mathrm{div}(\alpha_{l}u_{l}N_{i})=\alpha_{l}\left(B_{i}-D_{i}\right)-\alpha_{l}Z_{bi}-S_{i}$$

The transient solution to this equation is obtained by separating the transport and collision operators [24]. In the first step, the transport equation of the quantity *N_i_* is solved using a Finite Volume Method.

(4) $$\frac{\partial \alpha_{l}N_{i}}{\partial t}+\mathrm{div}(\alpha_{l}u_{l}N_{i})=0$$

In the second step, the population balance or Equation (5) is solved in each control volume applying the cell-average technique [22].

(5) $$\frac{\partial \alpha_{l}N_{i}}{\partial t}=\alpha_{l}\left(B_{i}-D_{i}\right)-\alpha_{l}Z_{bi}-S_{i}$$

The flotation kernel *Z_bi_* has been an issue of important studies in the literature and the reader will find details of the physical phenomena and applied model in References [25,26]. The aggregation term (*B_i_* − *D_i_*) is readily obtained through the use of the well-known Smoluchowski in Equation [17] where the expression of the collision frequency between two inclusion classes *i* and *j* is given by the equation below.

(6) $$Z_{ij}=\eta_{ij}\beta_{ij}N_{i}N_{j}$$

where $\beta_{ij}$ is the Smoluchowski collision kernel and $\eta_{ij}$ is the collision efficiency. The expression used for $\left(\eta_{ij}\beta_{ij}\right)$ stems from the modeling work performed at the mesoscopic scale of the inclusion (see Section 3.1 and Equation (8)).

Concerning the separation of particles induced by gravity, following the decomposition of particle velocity into local fluid velocity and Stokes’ velocity, the source term *S_i_* for the transport equation is shown below.

(7) $$S_{i}=\mathrm{div}(\alpha_{l}u_{s}N_{i})$$

where u_s_ is the vertical Stokes’ velocity in the case of small inclusions from whom Re*_p_* < 1.

Particular attention has been paid to inclusion entrapment at the liquid metal/slag interface. It is modeled following the approach based on a turbulent deposition law developed initially by Wood [27] for aerosols and adapted to hydrosols by Xayasenh [28]. The entrapment of inclusions at the ladle walls is not considered yet but has been found to be negligible [29].

## 3. Results and Discussion

### 3.1. Calculation of the Shear Rate Collision Kernel

Aggregation kinetics has been quantified through a collision efficiency (*η*), which is defined in Equation (2). Some simulation results for colliding pairs of particles are presented in Table 1. In typical ladle conditions, for turbulence dissipation rates lying between 10^−3^ and 10 m^2^/s^3^, the shear rate ranges between 30 and 3000 s^−1^, which yields Reynolds numbers between 10^−2^ and 1 for an inclusion around 10 µm in size.

The other parameter is the ratio between the diameters of the two particles. Particle density is kept close to the fluid density so that the Stokes number remains low. Particle inertia is negligible in these simulations. Moreover, particles undergo no other action than hydrodynamics (there are no distance forces between particles).

From Table 1, a conclusion can be drawn that there is little variation in the collision efficiency with the Reynolds number. Although cross-sections vary, which is illustrated in Figure 3, calculation of the efficiency results in similar values. Even at a Reynolds number much lower than 1, this efficiency remains quite significant (≈0.3) while the theoretical behavior in Stokes flow is particles avoiding each other at their initial relative positions, which leads to zero efficiency. However, the theoretical behavior at a high Reynolds number should be a collision efficiency of 1 since an inviscid fluid would induce no hydrodynamic interactions between particles. Therefore, they would follow their trajectories similar to the Smoluchowski’s model. Although counter-intuitive, this observation is consistent with collision efficiency observations from experimental studies in turbulent conditions [30].

This observation can be explained by flow recirculation patterns that demonstrate inertia in the liquid phase, which cannot be completely neglected when simulating particle interactions even at Reynolds numbers considered very low such as Re = 0.028. This is significant information since many numerical investigations in such conditions [19,31] use Stokesian Dynamics, which is unable to account for such effects. They, therefore, cannot be used for calculating inclusion aggregation kinetics.

While the collision efficiency is not sensitive to the shear rate, the particle size ratio has a significant impact for the conditions tested in this paper. This is primarily due to the low Stokes number of the considered particles since small particles are strongly deflected by the flow perturbation induced by larger ones, which favors avoidance when the size ratio increases.

Based on the above analysis and for the range of shear rates (i.e., turbulence dissipation rates) prevailing in steel ladle conditions, collision efficiency has been modeled with a very coarse relation that does not depend on Reynolds number but only on size ratio.

(8) $$\eta_{ij}=\max(0.4-0.1\;\frac{d_{p,\max}}{d_{p,\min}},0)$$

Although rather simple, this relation captures the two significant characteristics that have been extracted from simulations at a mesoscopic scale. Collision efficiency hardly depends on the shear rate and particle size that are both captured in the shear Reynolds number. It stays at about 0.3 for all flow conditions that inclusions experience in a steel ladle. However, it strongly depends on particle size ratio and it can be estimated by a linear fit for low size ratios. When size ratios increase, this coarse fitting law is not expected to apply, but since collision efficiency is very low, such size ratios do not play an important role in the overall aggregation process and do not require a finer description. 

### 3.2. Restructuring of Aggregates in a Pure Shear Flow

Ten aggregates made of 50 fully resolved primary spheres were simulated in plane shear flow conditions using the aforementioned LBM+IBM+DEM software. All aggregates had the same fractal dimension of 2.3 at the beginning of the simulations. Radii of gyration of the aggregates have been recorded over time and converted to fractal dimensions using an empirical correlation [16] since fractal dimension is a more common descriptor in aggregate studies. The shear Reynolds number for primary particles (Equation (1)) was kept small in all simulations (less than 10^−2^). Aggregate restructuring over time has been studied for different aggregate strengths relative to a hydrodynamic action by varying the magnitude of the normal and tangential forces between particles in contact. The scaling of the hydrodynamic force is achieved using the equation below. 

(9) $$F_{drag}=3\pi \mu \dot{\gamma }d_{p}^{2}$$

Then the dimensionless normal and tangential forces are expressed as the ratio of the corresponding force to the drag force expression from Equation (9).

As can be expected, dimensionless normal forces below 1 always led to aggregate breakage. However, as soon as the normal force is strong enough to hold the aggregate together, it seems to have very little impact on the aggregate morphology. The tangential force ratio, however, has a significant impact on the final structure of aggregates. Simulations with resolved hydrodynamic interactions show that aggregates of fractal dimension 2.3 tend to contract under shear conditions, which means their fractal dimension increases. Generally, the lower their contact tangential forces are, the greater their contraction is. However, the very different results between simulations with and without resolved hydrodynamics show that this aggregate behavior depends in a non-straightforward way on tangential forces. Higher tangential forces lead to stronger inter-particle bonds. However, stronger bonds increase the overall aggregate resistance to deformation and, therefore, favor bond breakage, which leads to a potential creation of new bonds.

**Figure 4:**
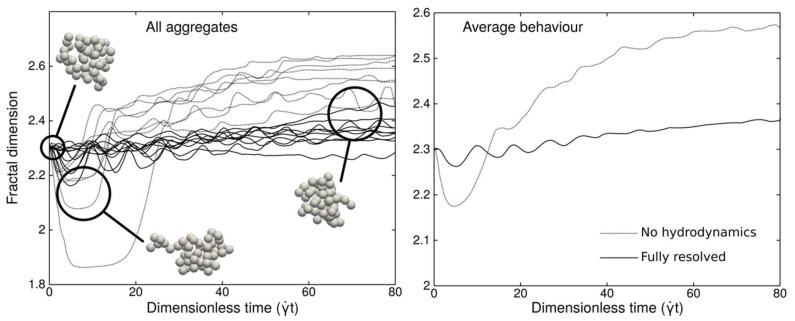
Evolution over time of the fractal dimensions of 50 particle aggregates. Dimensionless normal force is 1 and dimensionless tangential force is 0.1.

Figure 4 shows the evolution of the fractal dimension of all 10 aggregates with and without account for hydrodynamic interactions. In this example, a dimensionless normal force is 1 and a dimensionless tangential force is 0.1. When hydrodynamic interactions are not resolved, the LBM solver is disabled and the fluid action is modeled by a simple Stokes drag force and torque in the DEM using isolated particles in a plane shear. As expected, Figure 4 clearly shows that the hydrodynamics play a significant role in aggregate restructuring and they impact both kinetics and asymptotic trends.

All simulation results especially when accounting for hydrodynamic interactions show that the impact of restructuring on aggregate morphology is not strong enough to play a significant role on inclusion size evolution at the process scale. This size evolution is dominated by aggregation phenomena. However, the complex relation between tangential contact forces and bond breakage suggests that including varying aggregate breakage rates in the population balance may be a future significant step towards better modeling of inclusion behavior in metallurgical processes.

### 3.3. Macroscale Simulation

The simulation at the macroscopic scale of a metallurgical reactor is applied to an industrial 60 t steel ladle. Argon is injected through two porous plugs located at the base of the ladle. Two successive hydrodynamic conditions have been simulated including a first step with a relatively high stirring intensity corresponding to 70 NL/min of argon for each plug over 10 min (which is 5 times the mixing time), which is followed by a second stir for 10 min at a smaller argon flow rate (less than 10 NL/min for each plug). The model assumes that liquid temperature is uniform and equal to 1600 °C.

**Figure 5:**
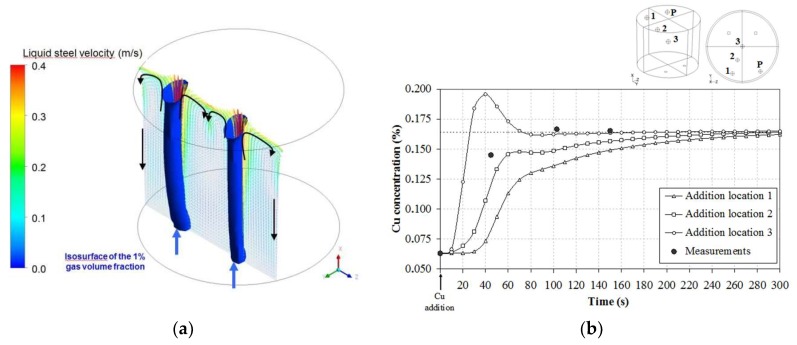
Hydrodynamic simulation of a 60 t steel ladle. (**a**) Predicted velocity of the liquid steel along with the argon plumes (isosurface of the 1% gas volume fraction) in a vertical plane passing through the porous plugs for a weak aeration. (**b**) Calculated Cu concentration profiles at the P sampling point compared with plant measurements for three platelet addition locations in the 60 t gas-stirred ladle.

The argon plume regions and the liquid steel velocity in a plane passing through the porous plugs of the ladle are shown in Figure 5a in the case of relatively weak aeration. One can observe the shapes of the two bubble plumes rising from the two porous plugs (the iso-surface of gas volume fraction equal to 1% is drawn). The turbulence, which mainly prevails in these regions, is characterized by a turbulent shear rate in the range of 150 to 1000 s^−1^. The liquid metal flow is associated with two recirculation zones with one in each half of the plane of symmetry. The liquid steel velocities are consistent with the magnitude found in the literature for equivalent industrial configurations [32].

#### 3.3.1. Hydrodynamic Validation

The hydrodynamic part of the numerical model was validated earlier using experimental data available in the literature. Physical modeling embodying aqueous as well as system with wood metal has been carried out to investigate the gas-stirred ladle system [33] used by many authors [34,35]. Comparisons between calculated and measured profiles of gas-retention *α_g_* and liquid velocity *u_l_* show a satisfactory prediction of the dispersion of the bubble swarm. Details of this first validation are available in Reference [7].

Another way to globally characterize the turbulent hydrodynamic behavior of the steel bath consists of introducing dissolved copper as a tracer and measuring the required time to full homogenization. Such a measurement of a characteristic mixing time can be easily compared to model predictions at an industrial scale, which involves completing the validation of the hydrodynamic simulations [36].

Copper platelets were added into the 60 t steel bath and samples were taken at time intervals of about 50 s. Since the location of platelet immersion and dissolution is not accurate, several simulations were conducted with varying points of copper platelets (1–3) and a fixed sampling location (P).

Figure 5b shows a relatively strong dependence of mixing time with initial tracer locations especially when the addition is simulated on the symmetry axis of the ladle. This, consequently, results in low values for mixing time compared to other addition locations. Although numerical simulations seem to underestimate the bath mixing time, the order of magnitude (2 min) agrees with the measurements. This mixing time cannot be regarded as negligible in comparison with the characteristic time of aggregation. Therefore, non-homogeneity of the inclusion population in an industrial ladle requires the development of 3D modeling.

#### 3.3.2. Evolution of the Inclusion Population in the Successive High and Low Stirring Steps

**Figure 6:**
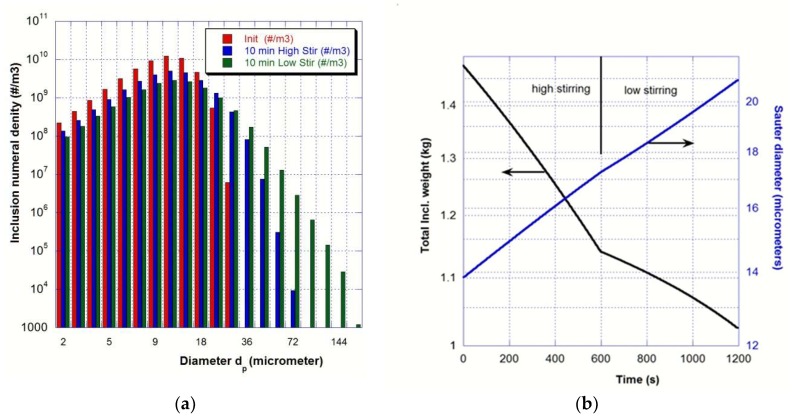
Average values of the non-metallic inclusion population in the ladle (**a**) PSD at three different times (initial in red, after 10 min of high stir in blue, and after 10 min low stir in green) (**b**) Time evolution of the total weight of non-metallic inclusions and of Sauter diameter.

According to the literature and more specifically the review proposed by Zhang and Thomas [1], a log-normal distribution has been used as the initial PSD with a total mass content equal to 0.176 kg/m^3^, which corresponds to 7.9 ppm of total oxygen content considering a population of calcium aluminate inclusions. This distribution matches relatively well with those analyzed in an industrial ladle for high steel quality since one kilogram of metal contains 5 × 10^6^ inclusions smaller than 20 μm but only 6 × 10^4^ larger than 20 μm. The initial distribution is plotted in red in Figure 6a where the inclusion population is rendered discretely into 20 different-sized classes. 

After 10 min of high stirring, the obtained PSD (in blue on Figure 6a) noticeably evolves with the birth of inclusions larger than 20 μm. The turbulent stirring occurred mainly in the bubble swarms leads to aggregation. Therefore, the shift of PSD toward larger size classes and the regular increase of the Sauter diameter *d_p_*_32_ (see Figure 6b). The reduction of the total weight of inclusions in the whole ladle observed in Figure 6b clearly highlights the effect of the removal mechanisms, which are flotation, deposition, and settling. One-third of inclusion content is then removed during the 20 min of treatment. The combination of aggregation with removal mechanisms leads to a noticeable decrease in the number of inclusions smaller than 20 μm, which is shown in Figure 6a.

The industrial practice usually consists in vigorous stirring to encourage particle collisions into larger ones followed by a “final stir” that slowly recirculates the steel to facilitate removal of large aggregates and to prevent any slag-free region of the steel surface and air re-oxidation. Although the developed turbulence is weaker, the 10 minutes of low stirring has a similar effect on the PSD (in green on Figure 6a) i.e., larger particles appear due to the aggregation of smaller particles leading to a fairly similar increase of the Sauter diameter *d_p_*_32_.

**Figure 7:**
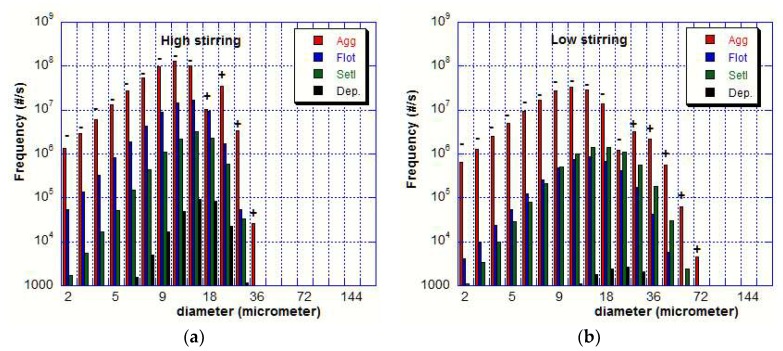
Frequency of mechanisms as a function of inclusion size (**a**) after 30 s of high stirring (**b**) after 30 s of low stirring. For aggregation, (−): reduced, (+): produced.

Numerical simulations allow us to compare the relative importance of the different removal mechanisms on the inclusion population. The frequencies of the aggregation, flotation, settling, and deposition events have been reported in Figure 7a (at the beginning of the high stirring process i.e., at time t = 30 s). As an example, the aggregation frequency *f_A_* is calculated by the equation below.

(10) $$f_{A}=\int_{V_{ladle}}(B_{i}-D_{i})dV$$

In the specific case of aggregation, signs (−) or (+) (sign of *f_A_* value) indicate that the numerical density of a given class decreases or increases.

Figure 7 clearly highlights that the major role is played by aggregation (although aggregation efficiency is much lower than 1, see Equation (8)) since its frequency is two orders of magnitude higher than the frequencies of flotation and settling for most size classes. This is the main reason why the PSD continuously evolves over time. The number density of inclusions smaller than 20 μm continuously decreases over time because of the aggregation mechanism and because of the removal phenomena (flotation and settling). Another obvious conclusion is the negligible role of the turbulent deposition of inclusions on the top surface of the ladle.

## 4. Conclusions

The split scale approach, which is the coupling process scale simulations with local inclusion dynamics through statistical models, has proven effective for capturing inclusion cleanliness trends during metallurgical processing. Inclusion cleanliness is mainly ruled by aggregation, which is observed in Section 3.3.2. Aggregation has an event frequency roughly two order of magnitudes larger than the one of the removal mechanisms. All inclusion removal mechanisms are strongly dependent on the size of aggregates and, therefore, conditioned by the aggregation kinetics. Therefore, the need for accurate aggregation models relate operating conditions to process efficiency. In this study, this need has been addressed by mesoscopic scale simulations from which quantitative information on aggregation kinetics has been extracted and captured in a statistical model. These simulations have shown very little variations with the particle size and shear rate for a broad range of operating conditions, which yielded constant aggregation efficiency for all of the same-sized particle pairs. However, this efficiency has been observed to drop drastically when increasing the particle size ratio. At the process scale, this means that the ruling aggregation kinetics are the ones between particles of the same size class.

## Figures and Tables

**Table 1:** Collision efficiencies (*η*) as a function of Reynolds number and the size ratio of the two inclusions.

Collision Efficiency		Reγ˙			
		0.028	0.071	0.213	0.319
d_p,1_/d_p,2_	1	$0.299_{\;-0.062}^{\;+0.070}$	$0.293_{\;-0.034}^{\;+0.024}$	$0.296_{\;-0.020}^{\;+0.021}$	$0.309_{\;-0.071}^{\;+0.076}$
	2				$0.197_{\;-0.037}^{\;+0.043}$
	3				$0.094_{\;-0.026}^{\;+0.027}$

## Nomenclature

Greek letters	
*α*	volume fraction
$\dot{\gamma }$	shear rate (s^−1^)
*η*	collision efficiency
*µ*	dynamic viscosity of the liquid metal (Pa·s)
υ	kinematic viscosity of the liquid metal (m^2^·s^−1^)
π	Archimedes’ constant
Latin letters	
*B*	population balance birth kernel (# m^−3^·s^−1^)
*D*	population balance death kernel (# m^−3^·s^−1^)
d	diameter (m)
*F*	force (N)
N	number of inclusions per unit volume of liquid metal (# m^−3^)
*f*	frequency (s^−1^)
*S*	gravity separation term in PBE (# m^−3^·s^−1^)
S	surface or surface area (m^2^)
*u*	fluid velocity (m·s^−1^)
V	volume (m^3^)
*y*	abscissa in the direction normal to the shear plane (m)
*z*	abscissa in the direction along the velocity gradient (m)
*Z*	collision frequency (m^−3^·s^−1^)
Subscripts	
A	relative to the aggregation mechanism
b	relative to gas bubbles and flotation
Col	relative to the actual collision cross section
drag	relative to the action of the liquid phase on the solids
*i, j*	discrete size classes in the population balance equation
g, l	gas or liquid phase
ladle	relative to the steel ladle
*p*	particle property
Smo	relative to Smoluchowski’s collision cross section
Compound	
Reγ˙	shear-based particle Reynolds number defined in Equation (1)

## References

[1] Zhang L., Thomas B.G. (2003). State of the art in evaluation and control of steel cleanliness. ISIJ Int..

[2] Kato T., Shimasaki S., Taniguchi S. (2010). Water model experiments for hydrodynamic forces acting on inclusion particles in molten metal under turbulent condition. Jim Evans Honorary Symposium: Proceedings of the Symposium Sponsored by the Light Metals Division of The Minerals, Metals and Materials Society (TMS).

[3] Xie Y., Orsten S., Oeters F. (1992). Behaviour of bubbles at gas blowing into liquid woods metal. ISIJ Int..

[4] Mendez C.G., Nigro N., Cardona A. (2005). Drag and non-drag force influences in numerical simulations of metallurgical ladles. J. Mater. Process. Technol..

[5] Liu H., Qi Z., Xu M. (2011). Numerical Simulation of Fluid Flow and Interfacial Behavior in Three-phase Argon-Stirred Ladles with One Plug and Dual Plugs. Steel Res. Int..

[6] Madan M., Satish D., Mazumdar D. (2005). Modeling of mixing in ladies fitted with dual plugs. ISIJ Int..

[7] De Felice V., Daoud I.L.A., Dussoubs B., Jardy A., Bellot J.P. (2012). Numerical modelling of inclusion behavior in a gas-stirred ladle. ISIJ Int..

[8] Li L., Liu Z., Li B., Matssra H., Tsukihashi F. (2015). Water Model and CFD-PBM Coupled Model of Gas-Liquid-Slag Three-Phase Flow in Ladle Metallurgy. ISIJ Int..

[9] Bellot J.P., De Felice V., Dussoubs B., Jardy A., Hans S. (2014). Coupling of a CFD and a PBE calculations to simulate the behavior of an inclusion population in a gas-stirred ladle. Met. Trans. B.

[10] Kwon Y.-J., Zhang J., Lee H.-G. (2008). A CFD-based Nucleation-growth-removal Model for Inclusion Behavior in a Gas-agitated Ladle during Molten Steel Deoxidation. ISIJ Int..

[11] Claudotte L., Rimbert N., Gardin P., Simonnet M., Lehmann J., Oesterle B. (2010). A Multi-QMOM Framework to Describe Multi-Component Agglomerates in Liquid Steel. AIChE J..

[12] Lei H., Nakajima K., He J.-C. (2010). Mathematical Model for Nucleation, Ostwald Ripening and Growth of Inclusion in Molten Steel. ISIJ Int..

[13] Eggels J.G.M., Somers J.A. (1995). Numerical simulation of free convective flow using the lattice-Boltzmann scheme. Int. J. Heat Fluid Flow.

[14] Sungkorn R., Derksen J.J. (2012). Simulations of dilute sedimenting suspensions at finite-particle Reynolds numbers. Phys. Fluids.

[15] Niu X.D., Shu C., Chew Y.T., Peng Y. (2006). A momentum exchange-based immersed boundary-lattice Boltzmann method for simulating incompressible viscous flows. Phys. Lett. A.

[16] Saxena A., Kroll-Rabotin J.-S., Sanders R.S. A numerical approach to model aggregate restructuring in shear flow using DEM in lattice-Boltzmann simulations. Proceedings of the 12th International Conference on Computational Fluid Dynamics in the Oil & Gas, Metallurgical and Process Industries.

[17] Smoluchowski M. (1916). Drei Vorträge über Diffusion, Brownsche Molekularbewegung und Koagulation von Kolloidteilchen. Z. Phys..

[18] Frungieri G., Vanni M. Dynamics of a Shear-Induced Aggregation Process by a Combined Monte Carlo-Stokesian Dynamics approach. Proceedings of the 9th International Conference on Multiphase Flow.

[19] Frungieri G., Vanni M. (2017). Shear-induced aggregation of colloidal particles: A comparison between two different approaches to the modelling of colloidal interactions. Can. J. Chem. Eng..

[20] Saffman P.G., Turner J.S. (1956). On the collision of drops in turbulent clouds. J. Fluid Mech..

[21] Becker V., Briesen H. (2008). Tangential-force model for interactions between bonded colloidal particles. Phys. Rev. E.

[22] Peglow M., Kumar J., Warnecke G., Heinrich S., Morl L. (2006). A new technique to determine rate constants for growth and agglomeration with size- and time-dependent nuclei formation. Chem. Eng. Sci..

[23] Ramkrishna D. (2000). Population Balances: Theory and Applications to Particulate Systems in Engineering.

[24] Toro E.F. (1999). Riemann Solver and Numerical Methods for Fluid Dynamics: A Pratical Introduction.

[25] Kostoglou M., Karapantsios T.D., Matis K.A. (2006). Modeling local flotation frequency in a turbulent flow field. Adv. Colloid Interface Sci..

[26] Mirgaux O., Ablitzer D., Waz E., Bellot J.P. (2009). Mathematical modelling and computer simulation of molten aluminium purification by flotation in stirred reactor. Metall. Mater. Trans. B.

[27] Wood N.B. (1981). A simple method for the calculation of turbulent deposition to smooth and rough surfaces. J. Aerosol Sci..

[28] Dupuy M., Xayasenh A., Waz E., Duval H. (2015). Analysis of non-Brownian particle deposition from turbulent liquid-flow. AIChE J..

[29] Lou W., Zhu M. (2013). Numerical Simulations of Inclusion Behavior in Gas-Stirred Ladles. Metall. Mater. Trans. B.

[30] Higashitani K.Y., Kiyoyuki K., Matsuno Y., Hosokawe G. (1983). Turbulent coagulation of particles dispersed in a viscous fluid. J. Chem. Eng. Jpn..

[31] Ren Z., Harshe Y.M., Lattuada M. (2015). Influence of the Potential Well on the Breakage Rate of Colloidal Aggregates in Simple Shear and Uniaxial Extensional Flows. Langmuir.

[32] Zhang L. (2006). Transport Phenomena and CFD Application during Process Metallurgy. Advanced Processing of Metals and Materials (Sohn International Symposium), Volume 4, New, Improved and Existing Technologies: Non-ferrous Materials Extraction and Processing.

[33] Xie Y.K., Oesters F. (1992). Experimental studies on the flow velocity of molten metals in a ladle model at centric gas blowing. Steel Res..

[34] Ling H., Li F., Zhang L., Conejo A.N. (2016). Investigation on the Effect of Nozzle Number on the Recirculation Rate and Mixing Time in the RH Process Using VOF plus DPM Model. Metall. Mater. Trans. B.

[35] Aoki J., Zhang L., Thomas B.G. Modeling of Inclusion Removal in Ladle Refining. Proceedings of the 3rd International Congress on Science & Technology of Steelmaking.

[36] Warzecha M., Jowsa J., Warzecha P., Pfeifer H. (2008). Numerical and Experimental Investigations of Steel Mixing Time in a 130-t Ladle. Steel Res. Int..

